# A Study on the Spatial–Temporal Evolution and Driving Factors of Non-Grain Production in China’s Major Grain-Producing Provinces

**DOI:** 10.3390/ijerph192416630

**Published:** 2022-12-10

**Authors:** Duan Ran, Zhanlu Zhang, Yuhan Jing

**Affiliations:** 1 School of Public Administration and Policy, Renmin University of China, Beijing 100872, China; 2 School of Marxism Studies, Chongqing University, Chongqing 400044, China

**Keywords:** non-grain production, spatial–temporal evolution, driving factors, hot spot analysis, spatial Durbin model

## Abstract

Food self-sufficiency in a large country with 1.4 billion people is very important for the Chinese government, especially in the context of COVID-19 and the Russian–Ukrainian conflict. The objective of this paper is to explore the spatial–temporal evolution and driving factors of non-grain production in thirteen major grain-producing provinces in China, which account for more than 75% of China’s grain production, using 2011–2020 prefecture-level statistics. In the present study, the research methodology included GIS spatial analysis, hot spot analysis, and spatial Durbin model (SDM). The findings of this study are as follows: (1) The regions with a higher level of non-grain production were mainly concentrated in the central and western regions of Inner Mongolia, the middle and lower reaches of Yangtze River and Sichuan, while the regions with a low level of non-grain production were mainly distributed in the Northeast Plain. The regions with a higher proportion of grain production to the national total grain production were concentrated in the Northeast Plain, the North China Plain, and the Middle and Lower Yangtze River Plain of China. The hot spot regions with changes in non-grain production levels were mainly distributed in the Sichuan region and Alashan League City in Inner Mongolia, and the cold spot regions were mainly distributed in Hebei, Shandong, Henan, and other regions. (2) An analysis of the SDM indicated that the average air temperature among the natural environment factors, the ratio of the sum of gross secondary and tertiary industries to GDP, the ratio of gross primary industry to the GDP of economic development level, the urbanization rate of social development, and the difference in disposable income per capita between urban and rural residents of the urban–rural gap showed positive spatial spillover effects. The grain yield per unit of grain crop sown area of grain production resource endowment, the total population of social development, and the area sown to grain crops per capita of grain production resource endowment all showed negative spatial spillover effects. The research results of this paper can provide a reference for the country to carry out the governance of non-grain production and provide a reference for China’s food security guarantee.

## 1. Introduction

Food security is an important issue related to social and economic stability and long-term national security. The development of urbanization and industrialization causes the transfer of agricultural labor to non-agriculture, the restructuring of rural industries, the decrease in the amount of high-quality cultivated land, the non-grain production of cultivated land, and other problems; thus, food security is constantly threatened [1,2,3,4,5,6].

As a country with a population of 1.4 billion people, the issue of food security has always been of concern to the Chinese government, which emphasizes that cultivated land is used for agricultural production and that high-quality cultivated land is used for grain production; studies have shown that the ratio of non-grain production to cultivated land in China reaches 27%, and different regions show different structural characteristics of non-grain production [7,8,9]. Two Chinese government documents, entitled “Opinions on preventing non-grain production of cultivated land and stabilizing grain production” and “Notice on resolutely stopping the non-agricultural behavior of cultivated land”, emphasize that cultivated land is used for agricultural production and high-quality cultivated land is used for grain production, and clarify the order of cultivated land use. The Chinese government opposes non-agricultural production on cultivated land and non-grain production on high-quality cultivated land. The Chinese government’s actions indicate stricter restrictions on cultivated land use for food security. Especially in the context of COVID-19 and the Russian–Ukrainian conflict, the Chinese government places greater emphasis on grain self-sufficiency [10]. Therefore, it is important to assess the level of non-grain production in China’s thirteen major grain-producing provinces.

Non-grain production on cultivated land refers to the agricultural production behavior of land operators who use cultivated land for non-grain cultivation, which can be divided into edible non-grain production and non-grain production of inedible production [8,11]. Edible non-grain production refers to the use of cultivated land for the cultivation of cash crops, such as peanuts, vegetables, and fruits, or for the development of efficient agriculture, such as livestock and poultry breeding; it mostly occurs in economically developed areas, urban suburbs, or areas with convenient transportation. Inedible non-grain production of cultivated land is the use of cultivated land for planting flowers and seedlings or for developing agroecological tourism, in pursuit of higher economic returns from the use of arable land [8,12,13]. Previous studies on the characterization of non-grain production have mostly used the ratio of cultivated area of non-grain crops to the total area of crops, the ratio of non-grain crops to the total area of cultivated land, etc. [14,15]. In this paper, the ratio of planted area of non-grain crops to the total crop area is used to express non-grain production.

Some scholars have conducted extensive and in-depth research on non-grain production. Most of the research has been on the development level, driving mechanism, externalities, and policy recommendations of non-grain production. In terms of the extent of the development of non-grain production in China, extant studies have shown that the phenomenon of non-grain production is becoming more and more serious, with more serious non-grain production in South, Northwest, Southwest, and Central China [8,16]. In terms of the mechanisms driving the phenomenon of non-grain production, some scholars have explored the analysis from the perspectives of cost and benefit [17,18], agricultural land transfer [19], industrial and commercial capital [20], policy understanding [21], farm household characteristics [22], labor transfer [23], cultivated land rental [24], and local government behavior [25]. Rising costs and declining returns of grain production [26], increased economic efficiency of non-grain economic crops [27,28], and dislocated government regulation [29] are important factors contributing to the serious phenomenon of non-grain production. In terms of the impact of non-grain production on cultivated land, different non-grain crops will have different impacts. Planting cash crops, such as vegetables and oilseeds, will have no impact on the cultivation layer, while planting economic forestry and fruit trees, dredging ponds for fish, and shrimp-rice symbiosis will cause damage to the cultivation layer to a certain extent [30,31,32]. According to several studies, non-grain cultivation may cause ecological issues, such as soil and water contamination [33,34,35]. In terms of policy recommendations for grain production, strengthening cultivated land protection [36], implementing structural control of non-grain crops [37,38], strengthening institutional construction [39,40], delineating functional zones for main grain production [41], establishing an early warning mechanism for non-grain production [40], providing compensation for cultivated land protection [42], implementing construction of cultivated land reclamation fund [8], encouraging sectoral collaboration [31], implementing a non-grain production property rights trading system [43], enhancing genetic improvement of crop varieties [44], regulating the use of transferred cultivated land [45], and improving grain-production conditions [46] and grain cultivation subsidies [47] can achieve stable grain production.

In summary, the following are some shortcomings of previous studies on non-grain production: (1) the research object has focused on the country or a particular province, without considering the non-grain production situation among the main grain-producing provinces; (2) fewer studies have integrated temporal and spatial evolution characteristics; and (3) fewer studies have considered spatial–temporal characteristics in the analysis of driving factors. Therefore, this paper takes the thirteen major grain-producing provinces in China as the study areas, uses spatial panel data from 2011 to 2020, and conducts a study on the spatial and temporal evolution trends and influencing factors of China’s non-grain production with the help of GIS spatial analysis, hot spot analysis, and spatial Durbin model, to provide a reference for China’s food security.

## 2. Materials and Methods

### 2.1. Study Area

**Figure 1:**
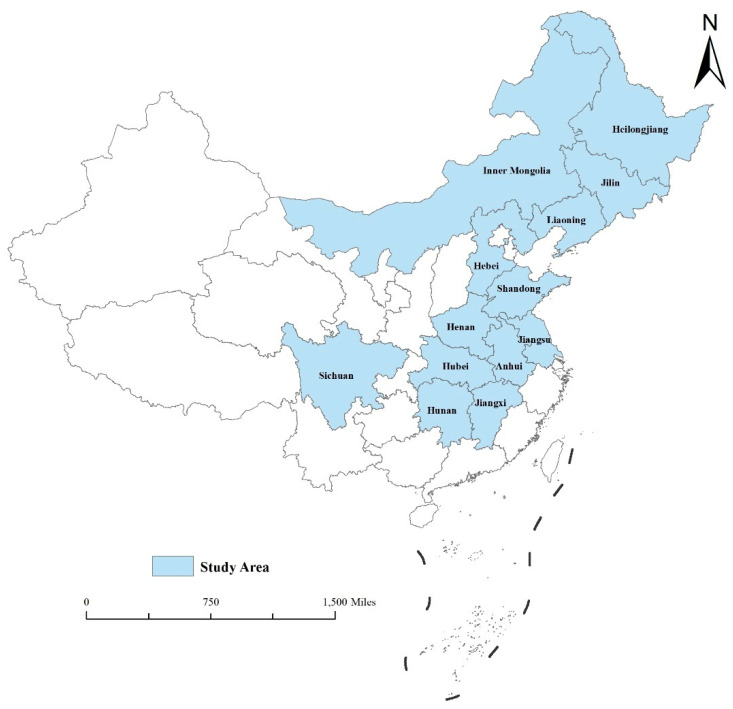
Location of the study area.

In this paper, thirteen provinces of Heilongjiang, Henan, Shandong, Jiangsu, Anhui, Sichuan, Jilin, Hebei, Hunan, Inner Mongolia, Hubei, Jiangxi, and Liaoning in China were used as the study areas (Figure 1). The data from China’s statistical yearbook show that these 13 provinces together account for more than 75 percent of China’s total grain production from 2011 to 2020. The structure of grain cultivation in these provinces has an important impact on China’s food security. Therefore, a study of non-grain production in these provinces is very necessary for national food security.

### 2.2. Data Sources

The data were collected from the following sources: (1) administrative boundary data from the National Basic Geographic Information Center (http://www.ngcc.cn/ngcc/, accessed on 5 June 2022); (2) several national and prefecture-level city statistical databases, including the “Statistical Yearbook,”, the “National Economic and Social Development Statistical Bulletin”, and the “Public Data of Government Departments”; and (3) DEM, temperature, and precipitation data from the Resource and Environmental Sciences and Data Center, Chinese Academy of Sciences (http://www.resdc.cn, accessed on 5 June 2022). Among them, slope data were obtained using DEM data and Arc Gis 16.0 processing. Missing values were added using the neighboring values, neighboring mean values, and EXCEL-trend function.

#### 2.2.1. Non-Grain Production Ratio

In combination with the research basis of its predecessors, we used previous research results to measure the level of non-grain production by the proportion of non-grain crops planted to the total area of crops sown, where non-grain crops are based on relevant Chinese laws and regulations and are non-grain crops other than cereals, soybeans, and tuber crops [15,48,49]. The formula can be written as follows:

(1) $$R=1-\frac{B}{A}\times 100\%\;$$

In Equation (1), *R* is the level of non-grain production, *B* is the area sown to grain crops, and *A* is the total sown area of crops.

#### 2.2.2. Share of Grain Production

We used the share of grain production of a region in the total national grain production as the degree of contribution of the region to the national grain production. The formula is as follows:

(2) $$G=\frac{P}{A}\times 100\%$$

where *G* is the share of grain production in a region in the total national grain production, *A* is the total national grain production, and *P* is the grain production of a region.

#### 2.2.3. Hot Spot Analysis

The hot spot analytical method can identify high- or low-value clustering areas of non-grain production changes, and it can effectively represent the spatial distribution characteristics of non-grain production changes [50,51]. In this paper, based on the hot spot analytical tool of the spatial analysis module of Arc Gis10.6 software, we used Getis-Ord Gi * to analyze the hot and cold spot distribution characteristics of statistical significance in the changes in non-grain production. Please refer to **Arc Gis 10.6 software** for the details of the formula.

#### 2.2.4. Spatial Durbin Model

According to the first law of geography, “Everything is related to everything else, but near things are more related to each other” [52]. Traditional OLS models do not consider spatial characteristics. Spatial panel models consider both the temporal and spatial characteristics of the samples simultaneously. Therefore, they are helpful for capturing the spatial–temporal heterogeneity of the statistical relationships between variables [53]. Spatial economic models mainly include the spatial lag model (SLM), the spatial error model (SEM), and the spatial Durbin model (SDM) [54]. Spatial lag models introduce the spatial lag terms of the explained variable as explanatory variables, and spatial error models introduce the spatial lag terms of the error term as independent variables [55]. While Spatial Durbin models introduce both the spatial lag terms of the dependent variables and the spatial lag terms of the error as independent variables [56]. Therefore, we used the spatial Durbin model to investigate the relationship between non-grain production and its influencing factors. The model is as follows:

(3) y=ρWy+Xβ+ε

(4) y=Xβ+μ,μ=λWμ+ε

(5) y=ρWy+Xβ+WXθ+ε

where *y* is the explained variable; *X* is the vector of the explanatory variable; *W* is the spatial weight matrix; *Wy* is the spatial lagged term of the explained variable; *β* is the coefficient of the explained variable; *ρ* and *λ* are the spatial autoregressive coefficients; *WX* is the explanatory variable spatial lag term; *θ* is the coefficient of the spatial lag term of the explanatory variables; and *ε* is the residual term. When *θ* = 0, the spatial Durbin model reduces to the spatial lag model; when *θ* + *ρβ* = 0, the spatial Durbin model reduces to the spatial error model.

The spatial Durbin model does not adequately respond to the effect of explanatory variables on the explained variables and does not take into account feedback effects. LeSage and Pace [57] proposed a partial differential approach to measure the spatial spillover effects of the variables, or the direct and indirect effects in their terms. Therefore, our study also used this approach. The spatial weight matrix was obtained using **GeoDa 1.20** software, and the spatial Durbin model was calculated using **Stata 16.0** software.

In this study, the non-grain production ratio was used as the explanatory variable. Concerning existing studies and data availability, 15 indicators were selected from natural conditions, resource endowment of grain production, agricultural science and technology level, urban–rural gap, agricultural production efficiency, social development, and economic development to perform the analysis of influencing factors (Table 1) [27,58,59,60].

In this study, both the explanatory variables and the explained variables were treated as natural logarithms to avoid the interference of the unit choice of economic variables on the test results. The value of the regression coefficient at this point indicates the elasticity of the explained variables to the explanatory variables, and the economic significance is the percentage change of the explained variables when the explanatory variables change by 1% [61].

## 3. Results and Discussion

### 3.1. Temporal Evolutionary Characteristics of Non-Grain Production

**Figure 2:**
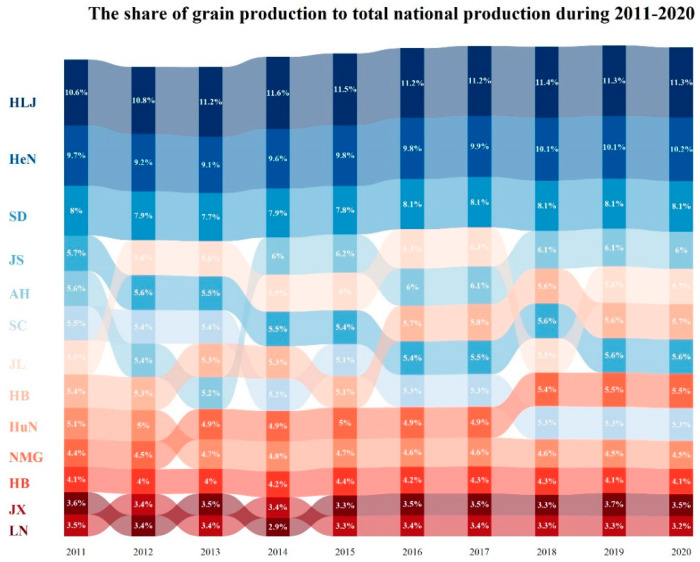
The share of grain production to total national production during 2011–2020.

From 2011 to 2020, as Figure 2 shows, Heilongjiang, Henan, and Shandong provinces were the major grain-producing provinces in China with the top three grain production ratios to the national grain production, with mean ratios of 11.20%, 9.75%, and 7.98%, respectively. Hubei Province, Jiangxi Province, and Liaoning Province had the lowest three grain production ratios to the national grain production, with mean ratios of 4.16%, 3.34%, 3.42%, respectively. Anhui Province, Jilin Province, Hebei Province, and Inner Mongolia showed an upward trend in fluctuations in the ranking of the ratio of grain production to total national grain production, while Jiangsu, Sichuan, and Hunan provinces showed a downward trend in fluctuations in the ranking of the ratio of grain production to total national grain production, and the remaining provinces showed a flat trend in the ranking of the ratio of grain production to total national grain production.

Table 2 shows that, from 2011 to 2020, the non-grain production ratio of China showed an upward trend in fluctuations, and the non-grain production ratios ranged from 28.69% to 30.28%; two of these years, 2019 and 2020, had non-grain production ratios exceeding 30%.

**Figure 3:**
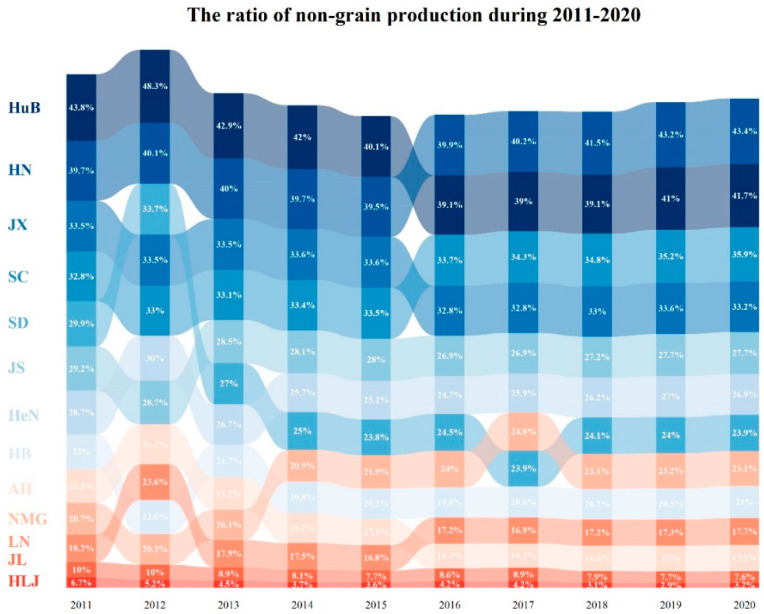
The ratio of non-grain production during 2011–2020.

Figure 3 shows that, from 2011 to 2020, the non-grain production ratios of the thirteen major grain-producing provinces showed a general downward trend, the non-grain production ratios of Sichuan and Hunan showed an upward trend, and the average non-grain production ratio in the southern provinces was higher than that in the northern provinces.

The non-grain production ratios in the southern provinces of Hubei, Hunan, Jiangxi, and Sichuan were always above 30%; the average non-grain production ratios in Hubei Province and Hunan Province were higher than 40%; and the average ratios of non-grain production in Jiangsu, Henan, Shandong, Inner Mongolia Autonomous Communities, Hebei, Anhui, and Liaoning provinces were 27.89%, 26.71%, 25.98%, 22.19%, 21.03%, 19.00%, and 18.03%, respectively. From 2011 to 2020, Heilongjiang and Jilin provinces ranked as the last two in terms of non-grain productivity: Heilongjiang’s average non-grain productivity was at 4.11%, with the highest non-grain production ratio at 6.68% in 2011 and the lowest non-grain productivity at 2.92% in 2019, showing a decreasing trend. Jilin’s average non-grain productivity was at 8.55%, with the highest non-grain productivity at 10.02% and the lowest non-grain productivity at 7.63 in 2020, showing a decreasing trend. Hubei Province and Hunan Province ranked alternately first and second in 2015 for non-grain production ratio; Jiangxi Province and Sichuan Province ranked alternately third and fourth in 2015 for non-grain production ratio except for 2012; Jiangsu Province, Henan Province, Inner Mongolia, and Liaoning Province ranked in a fluctuating upward trend for non-grain production ratio; and Shandong Province, Hebei Province, and Anhui Province ranked in a fluctuating downward trend for non-grain production ratio.

It could be observed that there was no significant relationship between the proportion of grain production to total national production and the ratio of non-grain production in the thirteen major grain-producing provinces during 2011–2020 (Figure 2 and Figure 3). For example, Heilongjiang had a high proportion of total national grain production and a low ratio of non-grain production; Henan and Shandong Province had a high proportion of total national grain production and a high ratio non-grain production; Jiangxi Province had a low proportion of total national grain production and a low ratio non-grain production; Hubei, Hunan, and Sichuan Provinces had a low proportion of total national grain production and a high ratio non-grain production.

### 3.2. Spatial Evolutionary Characteristics of Non-Grain Production

**Figure 4:**
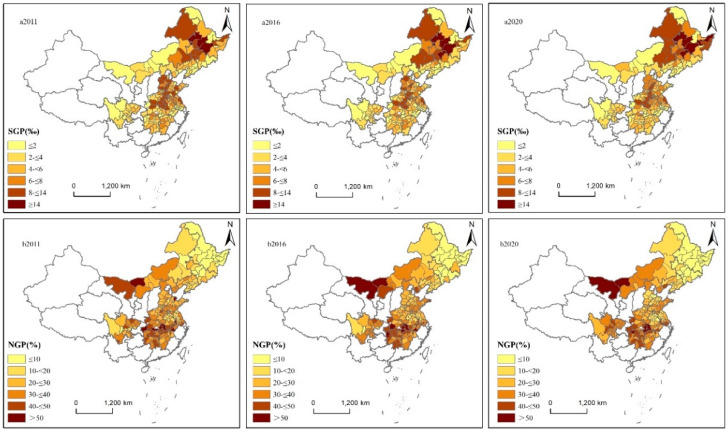
The ratio of non-grain production and the proportion of grain production to total national production in 2011, 2016, and 2020.

Figure 4 shows that the spatial distribution characteristics of the proportion of grain production to total national production and the non-grain production ratios in the thirteen major grain-producing provinces at the prefecture-level city scale were clearer. Due to space limitations, this paper only includes the spatial distribution of the proportion of grain production to the total national production and the ratios of non-grain production in 2011, 2016, and 2020. To better analyze, the proportion of grain production to total national production and the ratios of non-grain production are divided into six categories according to their patterns.

Figure 4a shows the national grain production for the years 2011, 2016, and 2020. The main grain-producing regions in China with a high proportion of national grain production were concentrated in the Northeast Plain, the North China Plain, and the Middle and Lower Yangtze River Plain, etc. Harbin, Qiqihar, and Suihua accounted for more than 14‰ of the national grain production, and Changchun and Jiamusi accounted for more than 14‰ of the national grain production in 2016 and 2020. In 2011, 2015, and 2020, the number of cities in the northeast region with a high proportion of total national grain production was on an increasing trend, while the number of cities in the middle and lower reaches of Yangtze River with a high proportion of total national grain production was in a diametrically opposite trend.

Figure 4b shows the ratios of non-grain production for the years 2011, 2016, and 2020. The spatial distribution characteristics of the non-grain production ratios were clearer. The regions with high non-grain production ratios were distributed in the central and western regions of Inner Mongolia and the middle and lower reaches of the Yangtze River, and the regions with high non-grain production ratios were distributed in the Northeast Plain. There were many regions where non-grain productivity was above 50%, especially in the regions such as Jingzhou City, Wuhan City, Huanggang City, Enshi Tujia, Miao Autonomous Prefecture, Ezhou City, Hubei Province, Dongying City Shandong Province, Nanjing City, Jiangsu Province, Bayannur City, and Inner Mongolia in 2011 (Figure 4b2011); Alashan League, Bayannur City, Inner Mongolia Autonomous Region, Wuhan City, Huanggang City, Ezhou City, Yichang City, Huangshan City, Xianning City, Hubei Province, Xiangxi Tujia, Miao Autonomous Prefecture, and Hunan Province in 2016 (Figure 4b2016); and Alashan League, Bayannur City, Inner Mongolia, Wuhan City, Ezhou City, Xianning City, Huanggang City, Huangshi City, Hubei Province, Zhangjiajie City, and Hunan Province in 2020 (Figure 4b2020).

### 3.3. Spatial–Temporal Characteristics of Changes in Non-Grain Production

**Figure 5:**
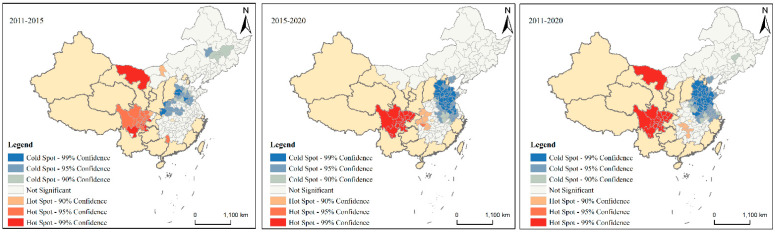
Hot spot analysis of non-grain production ratio changes in 2011–2015, 2015–2020, and 2011–2020.

This paper uses the difference in the proportion of non-grain production between the two periods before and after to characterize the change in non-grain production. Due to space limitations, the changes in the non-grain production ratios from 2011 to 2015, from 2015 to 2020, and from 2011 to 2020 are shown graphically using the hot spot analysis. Hot regions indicate high-value clusters for changes in non-grain production ratios and cold regions indicate low-value clusters for changes in non-grain production ratios. As shown in Figure 5, during the three study periods, the Sichuan region and Alashan League city in Inner Mongolia were hot spots of non-grain production rate changes, and Hebei, Shandong, and Henan regions were cold spots of non-grain production rate changes. From 2011 to 2015, the hot spot regions were distributed throughout Sichuan Province and a few scattered locations in western Hubei Province and northern Hunan Province, and the hot-cold regions were distributed in Shiyan City, Hubei Province, the southern region of Hebei, south-central Shandong, southern and northwestern Henan, and other regions (Figure 5, 2011–2015). From 2015 to 2020, the hot spots regions were distributed throughout Sichuan Province and western Hubei Province, with a few sporadic distributions in northern Hunan Province, and the cold spot regions were distributed in the whole area of Hebei except Chengde City and Zhangjiakou City, the whole area of Shandong except Weihai City and Yantai City, the whole area of Anhui Province except Chizhou City, the whole area of Jiangsu Province except Yancheng City, Nantong City, and Suzhou City, as well as a few sporadic distributions in Henan Province and Hubei Province (Figure 5, 2015–2020). From 2011 to 2020, the hot spot regions were mainly distributed in the whole area of Sichuan Province and the Alashan area in Inner Mongolia, and there were a few distributions in Hunan Province; the cold spot areas within Henan Province, Hubei Province, Hebei Province, and Shandong Province had little changes in distribution compared to the 2015–2020 period, with Nantong City and Suzhou City being added to Jiangsu Province and Huangshan City and Tongling City being added to Anhui Province (Figure 5, 2011–2020).

### 3.4. Driving Factors of Non-Grain Production

In this section, the first step is to determine whether there is a spatial correlation in the ratios of non-grain production, and the commonly used methods are Moran’s I index method, Geary index method, and Getis–Ord index method, etc. We used the widely used global Moran’s I index method to test whether there was a spatial correlation in the ratios of non-grain production, and the results are reported in Table 3. During the period from 2011 to 2020, the global Moran’s I results showed that there was a significant positive spatial correlation between the non-grain production ratios of the thirteen major grain-producing provinces in China; all Moran’s I indices were positive and all passed the 1% significance test. Therefore, we could use the spatial econometric models for driver estimation.

The second step is to determine which of the SLM, SEM, and SDM models to use. We used the Maximum likelihood (ML) method for estimations (Table 4). The statistic obtained using the Hausman test is 301.06, *p*-value = 0.000 (Table 5). Table 5 shows the results tested using Wald spatial lag and LR spatial lag, and all *p*-values are at the 5% level of significance. According to the above test results, the fixed-effect SDM is the final analytical model.

In Table 6, the direct effects on the non-grain production of a region are those exerted from the changes in the explanatory variables of the said region. The indirect effects on non-grain production are those exerted from the changes of explanatory variables in neighboring regions. The total effects are the sum of the direct and indirect effects.

Among the natural environmental determining factors, average height, average slope, average air temperature, and average rainfall were used to quantify the effects of terrain and climate, respectively, on non-grain production. The non-grain production of the regions was positively affected by average altitude, with no significant spatial spillover effects, and a significant weak positive direct effect (0.15321) and total effect (0.18885). Average slope had a substantial weak negative total effect (−0.16109) on the non-grain production of the regions, with no significant spatial spillover effects, and a significant weak negative direct effect (−0.14312) and total effect (−0.16109). The significant direct and total effects of average elevation and average slope showed that the former promoted regional non-grain crop production while the latter promoted regional grain crop production. This suggests a trend of grain crop production and non-grain crop production “up the hill,” similar to the changing pattern of cultivated land change in China. The average air temperature had a significant strong positive indirect (23.78788) and total effect (27.94924). This indicates a strong positive effect of average air temperature on non-grain production and a strong spatial spillover effect. This is because it is related to the spatial distribution characteristics of China’s temperature and non-grain production, which are low in the north and high in the south. Average rainfall had significant negative total effects (−0.22194). The significant negative total effect indicates that average precipitation promotes grain production.

The ability of a region to produce grain was gauged by its resource endowment for grain production. The grain production capacity of a region was measured using area sown to grain crops per capita and grain yield per unit of grain crop sown area. Area sown to grain crops per capita had significant negative direct (−0.53242), indirect (−0.28612), and total (−0.81854) effects on non-grain production. Grain yield per unit of grain crop sown area had a significant positive direct effect (0.17654), negative indirect effect (−0.49140), and negative total effect (−0.31486) on non-grain production. There were significant positive direct effects, negative indirect effects, and negative total effects of grain yield per unit of grain crop sown area. The above data illustrate that higher resource endowment for grain production has a significant spatial spillover effect on non-grain production. In other words, this indicates that regions with greater potential for grain production will encourage nearby regions to do the same. Of course, this applies to every region as well. However, the direct effect of grain yield per unit of grain crop sown area is to promote non-grain production in the region.

The total power of agricultural machinery per unit crop sown area was used to characterize the level of agricultural science and technology, which was used to indicate the degree to realize agricultural mechanization and scale. The significant direct (−0.21141) and total effects (−0.22252) showed that the region’s grain production benefited more from agricultural science and technology advancements than non-grain production.

The difference in disposable income per capita between urban and rural residents was used to characterize the degree of the urban–rural gap. The calculation results showed that the urban–rural gap had more significant direct (0.39398), indirect (0.23858), and total (0.63256) effects on non-grain production. This suggests that farmers in their region, in the neighboring regions, and in all regions are encouraged to produce non-grain crops because of the high profits they can generate.

The gross output value of farming was used to measure agricultural production benefits. The significant direct (0.50878) and total effects (0.52320) showed that a region’s and all regions’ non-grain production was positively impacted by agricultural benefits. This is due to China’s severe price regulations on grains, which are in contrast to those that apply to other agricultural products.

Urbanization rate and total population were used to characterize the degree of social development. Urbanization rate had significant positive indirect (0.47215) and total (0.51685) effects. The direct, indirect, and total effects of the total population were significant. This implies that the impact of urbanization rate on inter-regional non-grain production has spatial spillover effects. In other words, it means that an increase in urbanization rate in a region will boost non-grain production in the surrounding areas to meet the demand for non-grain products in the region. The total population had significant negative direct, indirect, and total effects, implying significant direct and spatial spillover effects on non-grain production in both a region and its neighboring regions. This indicates that the concentration of the population will have a great demand for grain production, which effect spills to the surrounding areas to increase the incentive to produce grain. Taken together, both the increase in urbanization rate and population agglomeration will have spatial spillover effects on grain production and non-grain production. In other words, this promotes grain production and non-grain production in the surrounding areas.

Among the factors influencing the level of economic development, there was no significant effect of GDP. However, the ratio of gross primary industry to GDP and the ratio of the sum of gross secondary and tertiary industries to GDP had significant positive direct (0.29213, 2.44747), indirect (0.52132, 1.75502), and total (0.81345, 4.20250) effects. In the context of strict control of grain prices, the ratio of the gross primary industry to GDP had a significant positive effect on direct and spatial spillover effects. In the context of urbanization and industrialization, farmers who remain in rural areas tend to grow non-grain crops of higher economic value to increase their income. Spatially, the ratio of gross primary industry to GDP and the ratio of the sum of the gross secondary and tertiary industry to GDP not only positively affected non-grain production in a region but also positively affected its neighboring regions to generate non-grain production. In other words, economic development in a region provides incentives for farmers in and around the region to engage in high-income non-farm work and to grow non-grain crops of high economic value.

## 4. Conclusions

This paper examines the spatial and temporal evolution and driving factors of non-grain production in thirteen major grain-producing provinces in China, using GIS spatial analysis, hot spot analysis, and spatial Durbin model. The main findings of this paper are as follows:

**(1)**  Non-grain production showed an upward trend from 2011 to 2020 in the thirteen major grain-producing provinces of China. From 2011 to 2020, Heilongjiang, Henan, and Shandong provinces were the top three provinces in the thirteen major grain-producing provinces of China in terms of grain production, with an average share of 11.20%, 9.75%, and 7.98% of the national grain production, respectively. Hubei, Jiangxi, and Liaoning provinces were the last three provinces in the thirteen major grain-producing provinces of China in terms of grain production, with an average share of 4.16%, 3.34%, and 3.42% of the national grain production, respectively. From 2011 to 2020, Hubei, Hunan, Jiangxi, and Sichuan provinces had a level of non-grain production above 30%, while Jilin and Heilongjiang provinces had a level of non-grain production below 10%. The share of grain production in the thirteen provinces was not significantly related to the level of non-grain production.
**(2)**  The regions with high ratios of grain production to total national production were concentrated in the Northeast Plain, the North China Plain, and the Middle and Lower Yangtze River Plain of China. The areas with high non-grain production were mainly concentrated in the central and western regions of Inner Mongolia, the middle and lower reaches of the Yangtze River, and Sichuan, while the areas with low non-grain production were mainly distributed in the Northeast Plain. The hot spot areas of non-grain production changes were mainly distributed in the Sichuan region and Alashan League City in Inner Mongolia, and the cold spot regions were mainly distributed in Hebei, Shandong, Henan, and other regions.
**(3)**  There was a significant spatial spillover effect of influencing factors in China’s major grain-producing regions, with regions being influenced by non-grain production in their neighboring regions. The analysis of the SDM indicates that the average air temperature of natural environmental factors, the ratio of the sum of gross secondary and tertiary industries to GDP, the ratio of gross primary industry to the GDP of economic development level, the urbanization rate of social development, and the difference in disposable income per capita between urban and rural residents of the urban–rural gap showed positive spillover effects and affected the non-grain production of neighboring areas. In addition, the grain production resource endowment of grain yield per unit of grain crop sown area, the total population of social development, and the area sown to grain crops per capita of grain production resource endowment all but significantly and negatively affected the non-grain production of neighboring areas.

## Figures and Tables

**Table 1:** Variables of non-grain production.

Factors	Variables	Definition	Unit
Natural environment	AA	The average altitude of a region	M
	AS	The average slope of a region	Degree
	AAT	The average air temperature of a region	K (Kelvin temperature)
	AR	The average rainfall of a region	Mm
Resource endowment for grain production	ASG	The ratio of sown area of grain crops to total population in a region	Hectare/person
	GYG	The ratio of grain yield to grain crop sown area in a region	Ton/ha
Agricultural science and technology	PAMC	The ratio of the power of agricultural machinery to crop sown area	kW/ha
Urban–rural gap	DIUR	The difference in disposable income per capita between urban and rural residents in a region	CNY
Agricultural production benefits	GOF	The gross output value of farming in a region	10,000 CNY
Social development	RU	The rate of urbanization in a region	%
	TP	The population in a region	10,000 people
Economic development	GDP	The GDP in a region	Billion CNY
	PGDP	The ratio of gross primary industry to GDP	%
	STGDP	The ratio of the sum of gross secondary and tertiary industries to GDP	%

**Table 2:** The non-grain ratios of China during 2011–2020.

Year	2011	2012	2013	2014	2015	2016	2017	2018	2019	2020
China	29.55%	29.43%	29.20%	28.89%	28.69%	28.58%	29.06%	29.45%	30.05%	30.28%

**Table 3:** Global Moran’s I of non-grain production from 2011 to 2020.

Year	Moran’s I	z	*p*-Value
2011	0.628	13.360	0.000
2012	0.599	12.727	0.000
2013	0.602	12.805	0.000
2014	0.623	13.263	0.000
2015	0.597	12.718	0.000
2016	0.631	13.444	0.000
2017	0.656	13.970	0.000
2018	0.674	14.344	0.000
2019	0.672	14.306	0.000
2020	0.675	14.354	0.000

**Table 4:** LM test.

Variables	SEM	*p*-Value	SLM	*p*-Value
Lagrange multiplier	716.051	0.000	672.099	0.000
Robust Lagrange multiplier	112.933	0.000	68.981	0.000

**Table 5:** LR, Wald, and Hausman test.

Test		Non-Grain Production	*p*-Value
LR test	Spatial error	150.64	0.000
	Spatial lag	85.21	0.000
Wald test	Spatial error	33.39	0.0008
	Spatial lag	24.96	0.0150
Hausman test		301.06	0.000

**Table 6:** Spatial spillover effects of variables.

Factors	Explanatory Variables	Direct Effect	Indirect Effect	Total Effect
Natural environment	AA	0.15321 ***	0.03565	0.18885 ***
		(0.0000)	(0.4613)	(0.0000)
	AS	−0.14312 ***	−0.01796	−0.16109 ***
		(0.0000)	(0.7550)	(0.0014)
	AAT	4.16136	23.78788 ***	27.94924 ***
		(0.1600)	(0.0000)	(0.0000)
	AR	−0.13728	−0.08466	−0.22194 ***
		(0.1229)	(0.4621)	(0.0019)
Resource endowment for grain production	ASG	−0.53242 ***	−0.28612 ***	−0.81854 ***
		(0.0000)	(0.0001)	(0.0000)
	GYG	0.17654 ***	−0.49140 ***	−0.31486 **
		(0.0016)	(0.0001)	(0.0190)
Agricultural science and technology level	PAMC	−0.21141 ***	−0.01112	−0.22252 ***
		(0.0000)	(0.8630)	(0.0007)
Urban–rural gap	DIUY	0.39398 ***	0.23858 *	0.63256 ***
		(0.0000)	(0.0965)	(0.0000)
Agricultural production benefits	GOF	0.50878 ***	0.01442	0.52320 ***
		(0.0000)	(0.9194)	(0.0006)
Social development	RU	0.04470	0.47215 ***	0.51685 ***
		(0.5230)	(0.0024)	(0.0014)
	TP	−0.45521 ***	−0.38623 ***	−0.84143 ***
		(0.0000)	(0.0013)	(0.0000)
Economic development	GDP	−0.07417	0.25401	0.17985
		(0.2550)	(0.1177)	(0.2936)
	PGDP	0.29213 ***	0.52132 ***	0.81345 ***
		(0.0000)	(0.0015)	(0.0000)
	STGDP	2.44747 ***	1.75502 ***	4.20250 ***
		(0.0000)	(0.0015)	(0.0000)

*p*-values in parentheses: *** *p* < 0.01, ** *p* < 0.05, * *p* < 0.1.

## Data Availability

The DEM, temperature, and precipitation data were obtained from http://www.resdc.cn. The data were also obtained from several national and prefecture-level city statistical databases, including the “Statistical Yearbook,”, the “National Economic and Social Development Statistical Bulletin”, and the “Public Data of Government Departments” (too many sites to list). The data that support the findings of this study are available from the first author upon reasonable request.
